# Value of CT-guided percutaneous needle biopsy of bone in the diagnosis of lymphomas based on PET/CT results

**DOI:** 10.1186/s40644-019-0230-8

**Published:** 2019-06-24

**Authors:** Zhiwei Wang, Haifeng Shi, Xiaobo Zhang, Jie Pan, Zhengyu Jin

**Affiliations:** Department of Radiology, Peking Union Medical College Hospital, Beijing, 100730 China

**Keywords:** Needle biopsy, Bone, Lymphoma, PET/CT

## Abstract

**Background:**

To evaluate the value of CT-guided percutaneous needle biopsy of bone in the diagnosis of lymphomas based on PET/CT results.

**Methods:**

A retrospective analysis of the records of all patients with percutaneous bone biopsies based on PET/CT results and a final diagnosis of lymphoma between January 2012 and August 2017 was performed. Thirty-one patients were included in this study. The success and complication rates were assessed.

**Results:**

The mean age of the 31 patients was 46.6 ± 21.2 years, and there were 16 men and 15 women. A definite diagnosis and accurate histological subtype were obtained in 26 patients, for a success rate of 84%. The most common subtype was diffuse large B cell lymphoma (*n* = 18). The remaining subtypes included three cases of marginal-zone lymphoma, two cases of follicular lymphoma, one case of Hodgkin’s lymphoma, one case of peripheral T cell lymphoma, and one case of B cell lymphoblastic lymphoma. No serious complications occurred in any of the patients.

**Conclusions:**

CT-guided needle biopsy based on PET/CT results is a reliable means of diagnosing and classifying lymphomas.

## Background

Lymphoma commonly occurs in the lymph nodes. However, every category of lymphoma can involve the skeletal system, with bone involvement observed in 16–20% of patients [1]. Pathological examination is fundamental to the accurate diagnosis of lymphomas, as the symptoms and imaging signs of lymphomas are highly heterogeneous [2–5]. Surgical biopsy is considered the standard for obtaining tissue for diagnosis. However, CT-guided percutaneous needle biopsy has become an important diagnostic tool due to its less invasive nature, shorter recovery time, and lower complication rate [6–10]. In recent years, a shift from open biopsy to CT-guided needle biopsy has occurred in the diagnosis and classification of malignant lymphomas [11–16].

Although CT-guided percutaneous needle biopsy has become the first step for acquiring tissue for the diagnosis of bone lesions [10, 17–24], this technique has been shown to be less efficient in diagnosing lymphoma of bone than other neoplasms and has not been widely used. 2-Deoxy-2-[18F] fluorodeoxyglucose positron emission tomography/computed tomography (FDG PET/CT) is increasingly used in cases of lymphoma and performs favourably in bone lesion detection compared to other imaging modalities [25–27]. We believe that CT-guided percutaneous needle biopsy of bone based on PET/CT results may improve the rate of lymphoma diagnosis. The aim of the study was to verify this hypothesis and to discuss the role and indications of this technique.

## Methods

Institutional review board approval was obtained for this retrospective study. A retrospective analysis of the records of all patients with percutaneous bone biopsies based on PET/CT results and a final diagnosis of lymphoma between January 2012 and August 2017 was performed. A total of 31 patients were identified. All patients with undiagnosed bone lesions were initially referred to our radiology department for CT-guided needle biopsy. All patients underwent a PET/CT examination for a systematic assessment within 2 weeks before biopsy.

### PET/CT procedure

Patients were instructed to fast for > 4 h before the PET/CT examination. Images of the whole body were acquired 40–60 min after 18F-FDG (0.15 mCi/kg) was injected. The PET/CT datasets were reconstructed automatically, and attenuation correction was performed with CT attenuation. The results were measured using the software on the PET/CT system workstation (TrueD, Siemens Medical Systems).

### Biopsies

**Fig. 1:**
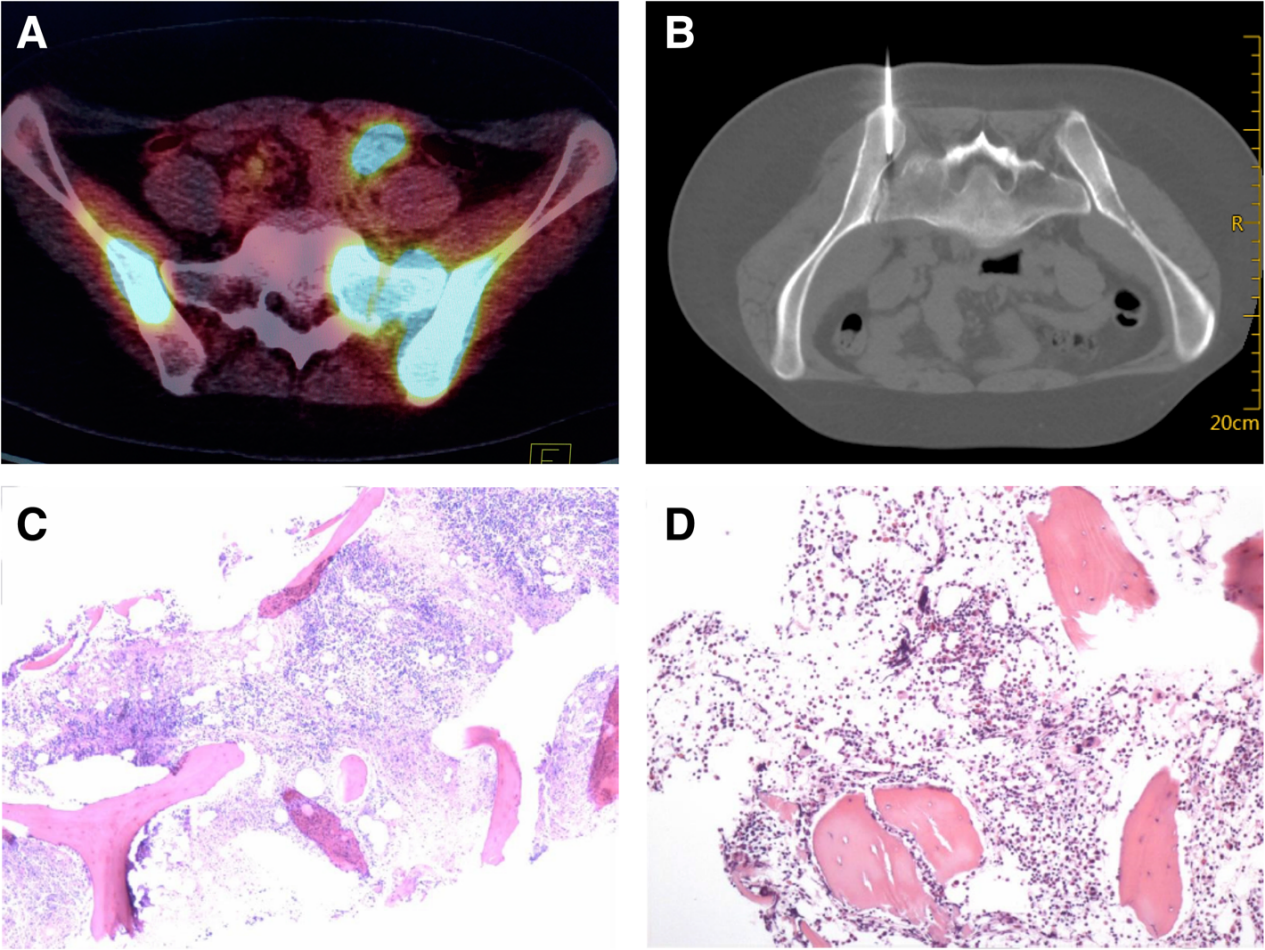
**a** PET/CT showed a hypermetabolic lesion in the left ilium of a 26-year-old woman. **b** Scan during biopsy of the left ilium showed the needle tip in the lesion. **c** Needle biopsy specimens (with H&E staining) showed lymphocytic infiltration, confirming the diagnosis of DBLB with immunohistochemical staining. **d** This patient also underwent blind needle biopsy of the left iliac crest (for a non-hypermetabolic lesion) in the department of haematology, and the specimen showed normal bone marrow

Before the biopsy, informed consent was obtained from all patients. All biopsies were performed under CT guidance. Patients were positioned in a prone or supine position depending on the bone lesion location. Patients underwent a CT scan of the area of the hypermetabolic bone lesions of interest based on the PET/CT results (Fig. 1a) using a spiral CT system (Sensation 16, Siemens, Germany or NeuViz 128, Neusoft, China). CT images were obtained at a slice thickness of 5 mm. The entry site was prepared and draped in a sterile fashion. All biopsies were performed under local anaesthesia consisting of an injection of 5–10 mL of 1% lidocaine hydrochloride. All biopsies were performed using a 13G bone biopsy needle (COOK, USA). The biopsy needle was inserted into the lesion. 5 millimetre sections were then obtained to verify the location of the needle tip (Fig. 1b). After the biopsy procedure, CT was immediately performed at the level of the biopsy site to search for the presence of complications, such as bleeding. Biopsy specimens were fixed routinely in 10% buffered formalin.

### Histological analysis

The lymphoma diagnosis was established by examining haematoxylin and eosin (HE)-stained biopsy sections and performing immunohistochemical staining, including for CD3, CD5, CD8, CD10, CD19, CD20, CD23, CD30, CD43, CD45, CD68, CD79a, AE1/AE3, Ki-67, epithelial membrane antigen (EMA), anaplastic lymphoma kinase (ALK), Cyclin D1, Bcl2, and Bcl6 (Fig. 1c). The diagnosis was reached by the consensus of at least two pathologists. The histological diagnosis was established according to the World Health Organization (WHO) classification of malignant lymphomas [28].

A biopsy was considered successful if a definite diagnosis and accurate malignant lymphoma classification could be established.

### Statistical analyses

Statistical analyses were performed using statistical software (SPSS, version 13.0, Chicago, IL, USA). To calculate *P*-values, Fisher’s exact *t* test was used. All *P* values of 0.05 and below were considered statistically significant.

## Results

The group of 31 patients included 16 men and 15 women, with a mean age of 46.6 ± 21.2 years (range, 16 to 85 years). Tissue samples of the metabolically active sites were obtained from all 31 patients for histological examination and immunohistochemical tests. The biopsy sites are shown in Table 1. Bony destruction was found at 19 biopsy sites on CT examination. The maximum SUV of the biopsy lesions was 8.6 ± 7.7.

**Table 1:** Clinical characteristic of the patients and bone lesions

Characteristics	Summary (*n* = 31)
Age^*^, Y (range)	46.6 (16–85)
Gender, n (%)	
Male	16 (51.6)
Female	15 (48.4)
Bone destruction, n (%)	
Yes	19 (61.3)
No	12 (38.7)
Bone marrow biopsy sites, n (%)	
Right humerus	1 (3.2)
Sternal handle	1 (3.2)
T3 vertebral body	1 (3.2)
T8 vertebral body	2 (6.5)
T11 vertebral body	1 (3.2)
T12 vertebral body	1 (3.2)
L2 vertebral body	1 (3.2)
L3 vertebral body	2 (6.5)
L4 vertebral body	1 (3.2)
S1 vertebral body	1 (3.2)
Left sacrum	1 (3.2)
Right sacrum	1 (3.2)
Left ilium	8 (25.8)
Right ilium	5 (16.1)
Left femur	2 (6.5)
Right femur	1 (3.2)
Right tibia	1 (3.2)
Pathology results, n (%)	
Needle biopsy results	26 (83.9)
Diffuse large B-cell lymphoma	18 (58.1)
Marginal zone lymphoma	3 (9.7)
Follicular lymphoma	2 (6.5)
Hodgkin lymphoma	1 (3.2)
Peripheral T-cell lymphoma	1 (3.2)
B cell lymphoblastic lymphoma	1 (3.2)
Surgical biopsy results	5 (16.1)
Marginal zone lymphoma	1 (3.2)
Diffuse large B-cell lymphoma	1 (3.2)
Peripheral T-cell lymphoma	2 (6.5)
Hodgkin lymphoma	1 (3.2)

The characteristics of the lesions we chose for biopsy are listed in Table 1. During each CT-guided core-needle biopsy, two to three specimens were obtained for satisfactory sampling (the total length of the samples obtained was 20–30 mm). There were 2 minor complications of haemorrhage. No serious complications were noted in any of the patients who underwent CT-guided needle biopsy.

A definite diagnosis and accurate histological subtyping were achieved by CT-guided needle biopsy in 26 out of 31 patients, for a success rate of 84%. The most common subtype was diffuse large B cell non-Hodgkin’s lymphoma (NHL) (*n* = 18). The remaining subtypes included three cases of marginal-zone lymphoma, two cases of follicular lymphoma, one case of Hodgkin’s lymphoma (HL), one case of peripheral T cell lymphoma, and one case of B cell lymphoblastic lymphoma.

Five patients with non-diagnostic needle biopsies were finally diagnosed by the surgical biopsy of other lesions. The final diagnosis in these cases is also listed in Table 1.

Six patients underwent blind needle biopsy of the iliac crest for non-hypermetabolic lesions in the department of haematology, and the specimens showed normal bone marrow (Fig. 1d).

We divided our patients into two groups based on the lesion characteristics of the biopsy site according to the CT examinations: the lesions in group 1 had bone destruction; the lesions in group 2 did not have bone destruction. The success rate of core-needle biopsy was 84% (16/19) in group 1 and 83% (10/12) in group 2, with no significant difference (*P* = 1.000).

## Discussion

Our retrospective study demonstrates the high accuracy of CT-guided needle biopsy in the diagnosis of lymphoma based on PET/CT results. Bone involvement in lymphoma is common and clinically relevant, as it has prognostic implications. Bone biopsy has its own advantages in lymphoma. The detection of lymphomatous cells in bone not only may aid in the diagnosis of lymphoma but can also be used as a staging tool in lymphoma, in which bone involvement indicates the highest disease stage [28].

In the past, surgical biopsy was considered the reference standard for obtaining tissue for the diagnosis of bone lesions. In recent years, image-guided percutaneous needle biopsy has become the first-line method for acquiring tissue for the diagnosis of bone lesions as a result of its less invasive nature, shorter recovery time, and lower complication rate [17–20]. However, the accuracy of image-guided bone biopsy is generally thought to be lower than that of soft tissue biopsy [18, 20]. Yang et al. evaluated 508 image-guided biopsies and found that bone lesions were more likely to have a non-diagnostic biopsy result than soft tissue lesions and were more likely to require a repeat biopsy [18]. Didolkar et al. evaluated 778 image-guided biopsies and found that bone biopsies had a higher non-diagnostic rate than soft tissue biopsies [20].

Furthermore, lymphoma is also considered the most likely condition to result in a non-diagnostic bone biopsy. Chang et al. retrospectively reviewed 963 consecutive CT-guided musculoskeletal biopsies and found that the diagnostic bone biopsy rate was significantly lower for lymphoma than metastases [22]. However, the CT-guided needle biopsy of soft tissue is a useful and reliable tool in the diagnosis and classification of malignant lymphoma. Balestreri L et al. retrospectively analysed 145 CT-guided needle biopsies of thoracic and abdominal soft tissue in 137 patients and found that a diagnosis was achieved in 96% patients with NHL [15]. Wang et al. retrospectively evaluated CT-guided core-needle biopsy in the diagnosis of primary pulmonary lymphoma and its subtypes and found that the success rate of achieving a definite diagnosis and accurate histological subtyping was 84% [11].

In our study, a definite diagnosis and accurate histological subtyping were achieved by CT-guided needle biopsy in 26 out of 31 patients, for a success rate of 84%. We consider the relatively high diagnostic rate to be due to two causes. First, we used PET/CT as a reference during biopsy. Guo et al. reported that PET/CT-guided percutaneous biopsy was an effective and safe method that yielded a high diagnostic success rate in the evaluation of hypermetabolic bone lesions in patients with suspected advanced lung cancer [29]. However, biopsy directly guided by PET/CT is still applied sporadically in clinical practice due to the high radiation dose, high cost and complex procedure. The main alternative to PET/CT guidance is CT-guided biopsy based on PET/CT results. Bitencourt et al. demonstrated that performing CT-guided biopsy based on PET/CT results was reliable, safe, and very accurate diagnostic method [30]. In this study, 95% of the biopsy procedures yielded conclusive results. PET/CT has become the standard method for assessing lymphoma and has improved the accuracy of staging [25, 26]. A meta-analysis of seven studies demonstrated a high specificity of PET/CT for the detection of bone involvement in diffuse large B cell lymphoma [31]. Our diagnostic rate was high for diffuse large B cell lymphoma based on PET/CT results.

Second, we used a relatively large (13G) bone biopsy system. This system is rapid, safe, and highly efficient for sampling and has been used as the most common tool for percutaneous CT-guided bone biopsy in our hospital. Guided by CT, a biopsy specimen approximately 2–3 cm in length is sufficient for immunohistochemical staining and complete subtyping. Some authors used 14-16G coaxial automated biopsy systems to perform bone biopsy [22]. The coaxial technique is useful for obtaining multiple biopsies in a single session. However, bone is a hard tissue, so it is difficult to adjust the angle of the external cannula for repeat biopsy, and the repeat biopsy specimen is not satisfactory in our experience.

Li et al. found that lytic bone lesions have a higher diagnostic yield by needle biopsy [21]. When we divided our patients into two groups according to the lesion characteristics, we found that the success rate of biopsy was not significantly different between the two groups. Therefore, the lesion characteristics do not seem to influence the success rate.

Limitations of this study include its retrospective nature and fairly small sample size of 31 patients, which limit the generalizability of our findings. This study did not include all subtypes of lymphoma. In certain disease subtypes, for example, diffuse large B cell lymphoma, the diagnostic yield of needle biopsy was high because the distribution of lymphomatous cells was fairly uniform. Therefore, the typical features of pathological lymphatic tissues are very important for the diagnosis of lymphoma. Five biopsies, in which lymphomatous cells were sparse or unevenly distributed, did not allow a definitive diagnosis to be reached and were considered unsuccessful.

## Conclusions

This study specifically assessed the usefulness of CT-guided percutaneous needle biopsy of bone in the diagnosis of lymphomas based on PET/CT results and demonstrates that percutaneous needle biopsy is a safe and accurate technique for providing a tissue diagnosis with a high yield.

## Data Availability

The datasets analysed in the current study are available from the corresponding author on reasonable request.

## Abbreviations

ALK: Anaplastic lymphoma kinase
EMA: Epithelial membrane antigen
FDG PET/CT: 2-Deoxy-2-[18F] fluorodeoxyglucose positron emission tomography/computed tomography
HE: Haematoxylin and eosin
WHO: World Health Organization
